# Supplementary material for: A MEKK1 – JNK mitogen activated kinase (MAPK) cascade module is active in Echinococcus multilocularis stem cells
Source: PLoS Negl Trop Dis. 2021 Dec 8;15(12):e0010027. doi: 10.1371/journal.pntd.0010027 (PMC8687709; doi:10.1371/journal.pntd.0010027)
Supplement: S1 Fig — (PDF) [file pntd.0010027.s002.pdf]

S1 Fig

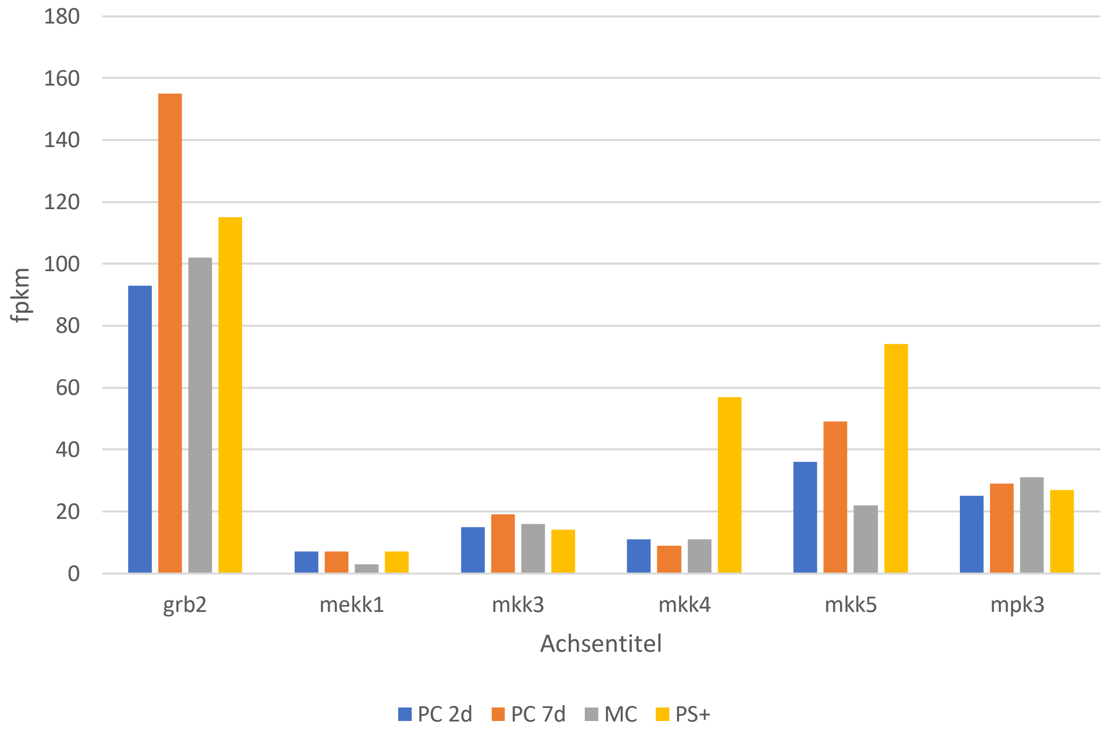

S1 Fig: Expression of *E. multilocularis* genes in larval stages. Depicted are the expression values of *emgrb2*, *emmekk1*, *emmkk3*, *emmkk4*, *emmkk5*, and *emmpk3* according to Next Generation transcriptomic analyses performed by [27]. Values are given as fragments per kilobase million (fpkm). The color code is shown below the graph for primary cells after 2 (PC 2d) and after 7 d (PC 7d) of incubation as well as metacestode vesicles without brood chambers (MC) and protoscoleces activated by incubation in pepsin/pH2. Please note that for each condition only one sample had been analysed.
