# Supplementary material for: A MEKK1 – JNK mitogen activated kinase (MAPK) cascade module is active in Echinococcus multilocularis stem cells
Source: PLoS Negl Trop Dis. 2021 Dec 8;15(12):e0010027. doi: 10.1371/journal.pntd.0010027 (PMC8687709; doi:10.1371/journal.pntd.0010027)
Supplement: S2 Fig — (PDF) [file pntd.0010027.s003.pdf]

S2 Fig

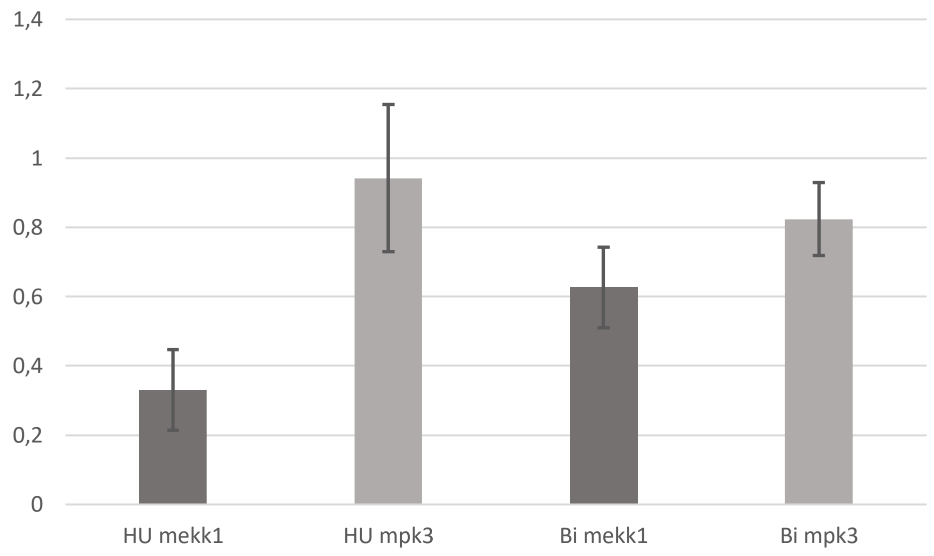

S2 Fig: RT-qPCR analyses for the expression of *emmekk1* and *emmpk3* in stem cell-depleted metacestode vesicles. RT-qPCR has been performed for emmekk1 (mekk1) and emmpk3 (mpk3) on metacestode vesicles treated with HU or Bi 25236 for 7 days. Expression values were measured in relastion to the constitutovely expressed control gene *elp* (EmuJ_000485800). Values for control vesicles (without inhibitor) were set to 1. Shown are the results of three independent experiments.
