# Supplementary material for: A MEKK1 – JNK mitogen activated kinase (MAPK) cascade module is active in Echinococcus multilocularis stem cells
Source: PLoS Negl Trop Dis. 2021 Dec 8;15(12):e0010027. doi: 10.1371/journal.pntd.0010027 (PMC8687709; doi:10.1371/journal.pntd.0010027)
Supplement: S3 Fig — (PDF) [file pntd.0010027.s004.pdf]

S3 Fig

```
EmuJ_000595100      ------------------------------------------------------------      0
TTK_HUMAN           MESEDLSGRELTIDSIMNKVRDIKNKFKNEDLTDELSLNKISADTTDNSGTVNQIMMMAN      60

EmuJ_000595100      ------------------------------------------------------------      0
TTK_HUMAN           NPEDWLSLLLKLEKNSVPLSDALLNKLIGRYSQAIEALPPDKYGQNESFARIQVRFAELK      120

EmuJ_000595100      ------------------------------------------------------------      0
TTK_HUMAN           AIQEPDDARDYFQMARANCKKFAFVHISFAQFELSQGNVKKSKQLLQKAVERGAVPLEML      180

EmuJ_000595100      -------------------------MGIYDYVPTNTGAWGNRL-------GKGPSLHK--      26
TTK_HUMAN           EIALRNLNLQKKQLLSEEEKKNLSASTVLTAQESFSGSLGHLQNRNNSCDSRGQTTKARF      240
                                               :     : :*: *:         .:* : :

EmuJ_000595100      -------------GFLPDLKER---LKPSVYG------FKSPDVQNES------------      52
TTK_HUMAN           LYGENMPPQDAEIGYRNSLRQTNKTKQSCPFGRVPVNLLNSPDCDVKTDDSVVPCFMKRQ      300
                                 *:   .*::     : . :*       ::***  : ::

EmuJ_000595100      ----------SNGEKSDSNVS-DI--SMEVVDSELQSPIRRPPWPLQRIGEEDEEAEAEL      99
TTK_HUMAN           TSRSECRDLVVPGSKPSGNDSCELRNLKSVQNSHFKEPLVSDEKSSELII----TDSITL      356
                                *.* ..* * ::    .* :*.::.*:        : *         .  *

EmuJ_000595100      ENRVESKK-TDMESLVTAQIPFQPSS----VQTFDESSCVHMSSQLSTSE----------      144
TTK_HUMAN           KNKTESSLLAKLEETKEYQEPEVPESNQKQWQSKRKSECINQNPAASSNHWQIPELARKV      416
                    :*:.**.  :.:*.    * *  *.*     *:  :*.*:: .   *:..

EmuJ_000595100      --DDSGAVTVQPGLVSVKSTLFLGRTGGVDKENVCQQPPPPPLSPSSSTRHRRV------      196
TTK_HUMAN           NTEQKHTTFEQPVFSVSKQSPPISTSKWFDPKSICKTPSSNTLDDYMSCFRTPVVKNDFP      476
                      ::. :.  ** :   *.:  :. :  .* :.:*: *    *.   *  :  *

EmuJ_000595100      ---SWAGPREALARLKKAETREMKVMEEAEADPISALVERSNVVVDGEKFIVLRQIARGG      253
TTK_HUMAN           PACQLSTPYGQPACFQQQQH-QILATPLQNLQVLASSSANECISVKGRIYSILKQIGSGG      535
                       . : *    * ::: :  :: .    : : :::   .. : *.*. : :*:**. **

EmuJ_000595100      FSSVFCVMNKKREMLALKRVGLVSASADVLEVCRNEVDLLLSLR-ESGRVIALYNYELSP      312
TTK_HUMAN           SSKVFQVLNEKKQIYAIKYVNLEEADNQTLDSYRNEIAYLNKLQQHSDKIIRLYDYEITD      595
                     *.** *:*:*::: *:* *.* .*. :.*:  ***:  * .*: .*.::* **:**::

EmuJ_000595100      SHLVMVLELAEQDLKSHLKMRQESGSLPDHVVTFLWNEMLACVKVIHDRRIVHLDLKPEN      372
TTK_HUMAN           QYIYMVMECGNIDLNSWLKKKKSI---DPWERKSYWKNMLEAVHTIHQHGIVHSDLKPAN      652
                    .:: **:* .: **:* ** ::.          .  *::** .*:.**:: *** **** *

EmuJ_000595100      FVIVRGMLKLIDLGISQRLPVDCTHMDLHKPMGSIVYMSPEQLSCIVGGKFASSNDGIDA      432
TTK_HUMAN           FLIVDGMLKLIDFGIANQMQPDTTSVVKDSQVGTVNYMPPEAIKDMSSSR---ENGKSKS      709
                    *:** *******:**::::  * * :  .. :*:: ** ** :. : ..:   .*.  .:

EmuJ_000595100      KVRLKTDVWALGVILYEMLHGRSPFGRTQ-QAAIMSAILSPTVSINFPHVDNAKLDEIYS      491
TTK_HUMAN           KISPKSDVWSLGCILYYMTYGKTPFQQIINQISKLHAIIDPNHEIEFPDIPEKDLQDVLK      769
                    *:  *:***:** *** * :*::** :   * : : **:.*. .*:**.: : .*::: .

EmuJ_000595100      HLLIP------------------VICRPT-------------------------------      502
TTK_HUMAN           CCLKRDPKQRISIPELLAHPYVQIQTHPVNQMAKGTTEEMKYVLGQLVGLNSPNSILKAA      829
                      *                    :  :*.

EmuJ_000595100      ----------------------------          502
TTK_HUMAN           KTLYEHYSGGESHNSSSSKTFEKKRGKK          857
```

S3 Fig: Amino acid sequence alignment of human and *Echinococcus* Mps kinases. Compared are the amino acid sequences of human Msp1/TTK (TTK_HUMAN; P33981), and the predicted sequence of the *Echinococcus* EmuJ_000595100 gene product. Sites of perfect alignment (*) as well as groups of strong (:) or weak (.) similarity are marked below the alignment. Residues for the interaction between Msp1 and SP600125 according to (Ref) are marked by a red arrow.
