# Supplementary material for: A MEKK1 – JNK mitogen activated kinase (MAPK) cascade module is active in Echinococcus multilocularis stem cells
Source: PLoS Negl Trop Dis. 2021 Dec 8;15(12):e0010027. doi: 10.1371/journal.pntd.0010027 (PMC8687709; doi:10.1371/journal.pntd.0010027)
Supplement: Attachment — Submitted filename: Response to Reviews.docx [file pntd.0010027.s005.docx]

**Manuscript PNTD-D-21-01208**

**Response to reviews.**

Below we address all critique raised by the reviewers point by point. For easier reading, we included the reviewer comments in italics (black) and our responses in red (no italics).

**Methods**

*Reviewer #1: The paper of Stoll et al. deals with molecular processes underlying the development of metacestode larvae of E. multilocularis that grow in a tumour-like manner within host tissues such as the liver. In this comprehensive and elegant study, the authors provide first evidence that a specific MAPK cascade is important in stem cells but also in differentiated cells of this parasite. Additional studies with a JNK inhibitor showed reduced metacestode vesicles and a reduction of proliferating stem cells in mature metacestode vesicles. This justifies speculations about the potential of JNK inhibitors as novel chemotherapeutics against echinococcosis.*

We thank the reviewer for the kind assessment of our work.

*Reviewer #2: (No Response)*

*Reviewer #3: (No Response)*

*Reviewer #4: this is a classical hypothesis driven research report, with clear objectives and experiments carried out with great expertise. The only question I have relates to the Echinococcus multilocularis isolates that are used. The authors indicate that they used E. multilocularis isolates(H95, GH09, Ingrid, J2012. . Are there differences between these isolates in terms of growth characteristics, protoscoleces formation or any other features such as virulence in mice?*

Yes, there are differences among the isolates. During long-term passage of *E. multilocularis* isolates within the peritoneum of jirds, they tend to lose the capacity of producing protoscoleces (~ after 5 years of passage, sometimes 1-2 years later). At the time when our experiments have been conducted, H95 (isolated in 1995), GH09 (2009), and Ingrid (2009) had already ceased protoscolex production (we call them PS- isolates). J2012 (2012) still produced brood capsules and protoscoleces, albeit at a reduced rate when compared to fresh isolates. During *in vitro* culture, PS- isolates produce larger vesicles (> 1 cm diameter) faster than PS+ isolates, which is particularly useful for WISH experiments (during repeated washing steps, larger vesicles don’t get lost when supernatant is taken off). In the case of inhibitor experiments, on the other hand, smaller vesicles are preferable since they can be kept in wells of low volume, thus reducing the amount of compound to be added. The production of large vesicles from PS+ isolates is time consuming since they usually start producing brood capsules at small size (less than 5 mm) and then only very slowly increase their volume (probably using all available energy for protoscolex production). For WISH experiments we therefore routinely use PS- isolates or PS+ isolates with somewhat reduced brood capsule production (like J2012) since these still can grow to vesicles sizes that are manageable during fixation/washing procedures. Differences among the isolates can also be observed *in vivo* with PS- isolates growing faster and less compact within the peritoneum of jirds whereas PS+ isolates produce more compact tissue blocks with smaller size (due to animal protective measures, we are not allowed to grow them *in vivo* for more than 3 months).

We now explain in more detail in the M&M section the characteristics of the isolates and specify for each experiment in the results section which isolate had been used.

--------------------

**Results**

*Reviewer #1: -yes*

*-yes*

*-yes*

*Reviewer #2: (No Response)*

*Reviewer #3: (No Response)*

*Reviewer #4: The analysis was done with great expertise, and figures clearly present the results. The authors show very nicely how the different components of this module interact with each other, and they also show that treatment with a JNK inhibitor led to reduced formation of metactodes from stem cells, as well as a specific reduction of stem cells in mature metacestodes. They indicate that JNK inhibitors could be used as novel chemotherapeutics against echinococcosis.*

We thank the reviewer for this kind assessment of our work.

--------------------

**Conclusions**

*Reviewer #1: -yes*

*-yes*

*-yes*

*-yes*

*Reviewer #2: (No Response)*

*Reviewer #3: (No Response)*

*Reviewer #4: The paper is very well written, experiments were done with great expertise, results are presented in a concise and clear manner, and most of the conclusions drawn from the results are sound. A few points to consider, especially in relation to the inhibitor studies:*

*SP600125: how specific is this inhibitor? The manuscript indicates that this inhibitor acts on metacestodes and germinative cells in the µM-range, while in mammalian cells this inhibitor works well at nM concentrations. What would be the normal concentration used for this inhibitor? Is there a risk that the massive amounts used to inhibit Echinococcus could produce off-target effects?*

The reviewer raises an important point. On the one hand, SP600125 is a widely used JNK inhibitor, not only in studies on mammalian JNK but also on invertebrate orthologs (see e.g. references on planarian JNK in the manuscript). It is also well known that SP600125 is > 300-fold more specific for JNK over Erk-kinase, p38-MAPK and numerous other (mammalian!) kinases (Bennett et al., 2001; Ref 52). Furthermore, at sub-lethal doses SP600125 phenocopied several phenotypes observed by JNK-specific RNAi in planarians, thus indicating that at least at concentrations up to 5 μM, there seem to be limited off-target effects in invertebrates (which express much less potential kinase targets than mammals). We thus believe that the effects on *Echinococcus* stem cells, which required lower concentrations of SP600125, specifically targeted EmMPK3, which, as outlined in the manuscript, contains all residues within the hydrophobic cleft that are involved in the interaction with SP600125 (line 558). Since we were still in the sub-lethal range when testing the inhibitor on metacestode vesicles, we also believe that at least the majority of effects can be attributed to EmMPK3 inhibition. Finally, in all tests involving metacestode vesicles, inhibitor concentrations are always quenched by the large amount of hydatid fluid within the cysts, which explains why we usually need lower inhibitor concentrations during tests on primary cells. Hence, it is not known whether 25 µM inhibitor given to metacestode vesicles really results in 25 µM at the target site.

Nevertheless, since there are several additional targets for SP600125 in mammals (Bain et al., 2003; Ref 53) we can, of course, not completely exclude off-target effects. As shown by Chu et al. (2008; Ref 54), the likeliest candidates for off-target effects are mitotic checkpoint kinases of the Mps family, of which one ortholog is expressed in *Echinococcus* (see manuscript text and Fig. S4). Whether SP600125 also targets the parasite Mps ortholog, will have to be elucidated in future studies.

We have added a new paragraph to the discussion section (lines 779-821) with detailed discussion on these points. We also added Fig. S4, showing the homology between mammalian Mps kinase and the *Echinococcus* ortholog.

*In relation to this comment, ideally the authors should show inhibitor studies carried out directly on EmMPK3 activity, not only on stem cells or whole metacestodes.*

Yes, we agree that such an assay would be informative. Indeed, we had previously established respective activity assays for EmMPK1 (Spiliotis et al., 2006; Ref 15) and EmMPK2 (Gelmedin et al., 2008; Ref 17). In the case of EmMPK2, which is a constitutively active MAPK, the enzyme could be directly (and easily) tested after recombinant expression in *E. coli* (Gelmedin et al., 2008). In the case of ‘normal’ MAPKs, which have to be activated by upstream MAPKK, the assay is much more difficult. For EmMPK1, we had a specific antibody (commercially available against human Erk kinase) to immunoprecipitate the enzyme from *Echinococcus* lysate (Spiliotis et al., 2006). In the case of EmMPK3, however, no such antibody is available. We had tested mouse monoclonal antibody G-7, directed against phospho-JNK, but in contrast to similar experiments for EmMPK1 and EmMPK2, where only one band was detected in *Echinococus* lysate, we detected multiple bands for G-7, thus preventing specific immunoprecipitation of EmMPK3 from lysates. Finally, recombinantly expressed EmMPK3 (just like other JNKs) cannot ‘artificially’ be activated e.g. by introducing phospho-mimetic mutations (at least such an assay has not been published). Hence, it is not possible to come up with a EmMPK3 activity assay within a reasonable time frame. Nevertheless, based on sequence homologies (and conserved residues within the drug binding pocket), EmMPK3 is the likeliest target for SP600125.

*- Based on the efficacy of the inhibitor, I strongly believe that the statement that JNK inhibitors could be candidates for chemotherapeutical treatment is not really supported by the data.*

We agree in so far as SP600125 is surely not a promising drug. This does not mean, however, that it is impossible to generate inhibitors which bind more specifically to *Echinococus* EmMPK3 than to human JNK. At least SP600125 has been specifically developed (or better: selected) to bind to human JNK and does so probably better than to invertebrate orthologs. It should thus be possible to also develop inhibitors which bind more specifically to invertebrate JNKs (including *Echinococcus* EMPK3) if the parasite enzyme is taken as primary target for drug selection. We nevertheless modified any respective statements in the abstract and the discussion section (lines 45-46; lines 748-751) to clearly indicate that a selection process still has to be made if EmMPK3 (or other components of the *Echinococcus* JNK cascade) should be used as a target. We hope the reviewer can agree to these modifications.

--------------------

**Editorial and Data Presentation Modifications?**

*Reviewer #1: Major comment*

*In their EdU analyses, the authors observed a difference between the isolates Ingrid and J2012 (text and Fig 2B). Can the authors speculate about a presumptive reason of this difference? Could this have something to do with the origin of these isolates, is there a different genetic equipment?*

As outlined above (response to reviewer #4), there are differences among the isolates, particularly concerning the ability of protoscolex production and vesicles sizes during *in vitro* culture. We have already extensively searched for genetic differences among the isolates which would explain these features, but did not find any so far (we assume that as yet unknown epigenetic mechanisms are involved). In the case of our experiments, however, we propose a much simpler, experimental reason to account for these differences. Since the vesicles we use for WISH on isolate J2012 are routinely much smaller than those we can use for PS- isolates, they also contain much less hydatid fluid which can quench EdU incorporation (as already discussed previously (Koziol et al., 2014; Ref 2) we have to use quite high concentrations of EdU on *Echinococcus* metacestode vesicles since much of the nucleotide analogue is diluted in hydatid fluid). Hence, we propose that the hydatid fluid of larger vesicles (e.g. of isolate Ingrid) influences the final concentration of EdU to which stem cells have access so that many of them fall below the detection threshold. In the case of smaller vesicles (such as of isolate J2012) this effect is diminished, leading to higher rates of EdU positive cells. Of course, we cannot exclude that there are some slight differences in stem cell proliferation rates among isolates, but we consistently see an inverse correlation between vesicle size used for WISH and the number of EdU positive cells. In the revised version of the manuscript, we now provide further explanation on these peculiarities of the experimental system (lines 380-388).

*Minor comments*

*line 55: In this work, …*

*line 58: … in parasite stem cells, which drive …*

*line 74: … infectious eggs, which are released by the definitive host and are orally taken up by the intermediate. (Here and elsewhere, insert commas in front of “which”)*

*line 94: During recent years, we have …*

*line 156: Insert space between …J20212) and [29]…*

*line 161: define (d) for days and use abbreviation afterwards.*

*line 188: define WMISH here (to my knowledge, whole mount in situ hybridization is abbreviated as WISH) and use only the abbreviation later on (see e.g. Fig. 6 legend)*

*line 193 following: hours (h). Use abbreviation afterwards.*

*line 309: delete “could”; better: … we detected …*

*line 350: delete the second “been”*

*line 359: delete “well”*

*lines 361/367: axins*

*line 377: In the legend of Fig 3, Y2H has been defined as abbreviation. This should be done in the text upon first use of the term yeast two hybrid.*

*line 401: delete “could”; better: … we identified …*

*line 409: Echinococcus should be given in italics (please check italics writing for species names throughout the manuscript; see e.g. line 517: Drosophila)*

*line 514: We next investigated …*

*line 515: ATP-competitive*

*line 542: italics writing for „in vitro“ should be harmonised throughout the manuscript*

*line 577: In this work, …*

*line 629: delete “could”; better: By Y2H, we observed …*

*References: mixture of upper and lower case use for paper titles, please harmonise.*

In all points listed above, we modified the manuscript exactly as suggested by the reviewer.

*Reviewer #2: (No Response)*

*Reviewer #3: (No Response)*

*Reviewer #4: No comments*

--------------------

**Summary and General Comments**

*Reviewer #1: The manuscript is well written, and its content perfectly fits to the aims and scope of PLoS NTD. As such, it will be of high interest for the community. I have only little to critisize.*

We thank the reviewer for this assessment.

*Reviewer #2: This paper presents another important advance from this lab in the understanding of signal transduction and growth in tapeworms. It is well written and clearly presented (albeit so littered with acronyms in places it can become almost non-prose) and the methods and interpretations are sound. I would like to have seen the genome-wide effects of the inhibition assays via transcriptome profiling, but there is more than enough functional and other evidence to support their stated conclusions and hypotheses. I recommended it highly for publication.*

We thank the reviewer for this assessment.

*Minor comments and corrections:*

*81. This is a characteristic of all flatworms – why not state that, unless to leave the impression it’s unique to E. mul. or some other exclusive group of flatworms?*

We modified the manuscript accordingly.

*166. I was immediately wondering why there was no discussion of how were GOIs chosen/identified – however, reading further I found this was discussed in detail in multiple places. Perhaps here you could mentioned that fact – eg. “Identification of GOIs is discussed in conjunction with results below.”*

We modified the manuscript accordingly.

*266 WormBase*

*325. italicise emmekk1*

*331. This final conclusion should be joined to the end of the preceding paragraph.*

*390. Add parentheses around “*” and “:”*

*409. Italicise Echinococcus*

*517. Italicise Drosophila*

*556. As above, this final conclusion should be joined to the end of the preceding paragraph (only paragraphs are offset from other paragraphs – and by definition a paragraph consists of > one sentence)*

All points listed above were modified exactly as suggested by the reviewer.

*608. A figure of the proposed signalling cascade would be helpful*

We included novel Fig. 8 in the revised version showing the proposed, combined signalling cascades analysed here and in previous work of our group.

*643. Italicise Echinococcus*

We did so.

*663. Transcriptome profiling of these assays would have been incredibly informative for tracking the full downstream affects of the inhibition.*

Yes, we agree that this would be informative, but we believe that we have already presented sufficient data in this work. We will consider it in future analyses.

*Happy birthday KB!*

KB thanks for these greetings.

*Reviewer #3: This manuscript describes the characterization of MEKK1/JNK signaling cascade in E. multilocularis, the last module of the MAPK cascades that has not been identified yet in the parasite. The authors identified key members of the JNK signaling pathway and showed that the JNK inhibitor SP600125 inhibits the formation of metacestode vesicles from stem cells and reduces the number of proliferating cells in vesicles, suggestive a role of the JNK signaling in regulating E. multilocularis stem cells. The manuscript is well presented and well written. Overall, I endorse this work but do have a few concerns and suggestions.*

*Major concerns:*

*1) The statements that the authors addressed in Abstract “that are most likely germinative cell progeny” and L331-333 “these analyses indicated that emmekk1 is expressed …”.*

*I’m afraid that there is lack of strong evidence to conclude that the emmekk1+EdU- (EmMPK3+EdU-) cells are immediate stem cell progeny. I think the best way to obtain direct evidence is using a sequential EdU/BrdU pulse staining followed by WMISH, which is, however, technically difficult in the parasite so far. Alternatively, the authors, to make this issue clearer, may check the emmekk1+ (EmMPK3+) cells in the vesicles after a long period of time of EdU incubation (e.g. 24 h) or after different time of EdU incubation (e.g. 5-24 h), or after a treatment with hydroxyurea which eliminates most germinative cells. Otherwise, I suggest the authors should soften this conclusion accordingly in the results and discussion.*

The reviewer is right. Since we only had 30 days to resubmit, we decided to soften our statements. We have deleted all allusions to stem cell progeny and call the EdU-/*emmekk1+* as well as the EdU-/*emmpk3+* cells ‘differentiated and/or differentiating’ cells.

*2) L374-375: “indicated that EmMEKK1 could act as a signal transducer of EGF signaling in E. multilocularis via interaction with EmGrb2”*

*These results only indicate that EmMEKK1 interacts with EmGrb2, however, whether the interaction responds to EGF signal remains largely uncertain in the parasite. I suggest the authors tone down their statement on this.*

The reviewer is right. We changed the wording to ‘acts as a signal transducer downstream of EmGrb2.’

*Others:*

*1) The authors found that the vesicles lost their structural integrity after 6-13 days of SP600125 treatment (Fig.7). This is an interesting result. It was shown that after specific elimination of stem cells by hydroxyurea the vesicles usually remain structurally intact for at least two weeks. A similar result was also observed by the same group when using the PLK1 inhibitor BI2536, which targets specifically the mitotic cells. Taken together, these results suggest that inhibition of the JNK signaling by SP600125 may not only affect the stem cells, and/or that SP600125 may have targets other than JNK in the parasite. I suggest the authors could make some discussion on this.*

The reviewer is right. We changed the discussion accordingly and now explicitly state that SP600125 might also act on differentiated cells, thus explaining why the vesicles lost structural integrity earlier than in the case of inhibitors which only target stem cells (see lines 788-796 of the revised manuscript).

*2) Fig.7. The title for y-axis in Fig.7B. Is it the percentage of EdU+ cells of total cells in the vesicles? The ratio for ctrl is > 15%, much higher than those as shown in Fig.2 and 6 (~8%). In addition, did the authors check the number of EdU+ cells and the expression of some proliferation markers (e.g. h2b) in the vesicles after a short term of treatment with SP600125 (24-72 h)? According to the results presented here, it seems that, in spite of the high expression of EmMPK3 in ~2/3 of the proliferating stem cells, SP600125 impairs the maintenance rather than the proliferation of the stem cells.*

Yes, the percentage of EdU+ cells in these vesicles was much higher than in the WISH experiments. As already outlined above (responses to reviewers #1 and #4), we routinely use rather small vesicles for inhibitor experiments to minimize concentrations of compound to be added. At smaller vesicle size, however, the quenching effect of hydatid fluid on EdU incorporation is also minimized (see discussion above), which would explain why we see higher percentages of EdU positive cells. Apart from that, we also used isolate H95 for these experiments which, as PS- isolate that is in intraperitoneal passage since 1995, is proliferating quite well when compared to younger isolates. We added this information to the results section.

No, we did not perform experiments using *h2b* as a marker. We agree that this would be informative and thank the reviewer for the suggestion. However, as we had previously reported (Koziol et al., 2014; Ref 2), WISH on *Echinococcus h2b* is indistinguishable from patterns that we see on EdU incorporation (both are markers for S-phase), so we only used EdU incorporation to identify proliferating stem cells. We did, however, perform short term experiments concerning EdU incorporation and, at least after 72 h, did not observe a statistically significant reduction of EdU positive cells (now incorporated in the results section lines 648-651). Additional experiments are surely necessary to distinguish between effects on stem cell maintenance or proliferation. Similar to what had been observed for planarians, we do speculate that also in *Echinococcus* the transition of G2/M phase is affected. All this is part of the discussion (lines 786-791).

*3) The authors quoted the transcriptome data (ref. 27) several times to indicate the gene expression. However, I think it is better to perform q-PCR experiments to verify this, especially for those genes of which expression is high in the stem cell populations.*

We did not perform RT-qPCR experiments on the expression of *emmekk1* and *emmpk3* in metacestode vesicles since (a) we could easily amplify the respective transcripts from metacestode vesicle cDNA preparations by regular PCR, thus indicating expression, and (b) because we performed WISH for both genes, which we consider even more informative. For reasons of clarification, we included in the revised version a new figure (Fig. S2) showing the fpkms of all new genes analysed according to previously generated transcriptome data (Tsai et al., 2013).

We nevertheless thank the reviewer for the suggesting Rt-qPCR studies since particularly concerning the discussion whether *emmekk1* and/or *emmpk3* are expressed in stem cell progeny, we considered respective experiments on metacestode vesicles after elimination of stem cells via HU or Bi2536 treatment very informative. For the revised version of our manuscript, we thus performed these additional experiments. To fully eliminate stem cells for metacestode vesicles, treatment with HU (hydroxy urea) or the Polo-like kinase inhibitor Bi2536 for ~7 days is necessary (Koziol et al., 2014, Schubert et al., 2014) and, thus, most probably not only the stem cell compartment is affected but also immediate progeny. Interestingly, in the case of *emmekk1*, we see a statistically significant reduction of transcripts in both HU and Bi2536 treated vesicles when compared to control vesicles, whereas no such effect was observed for *emmpk3*. In the case of *emmekk1*, these results indeed point to expression in stem cell progeny (in addition to germinative cells). However, since we have not yet performed pulse-chase experiments, we left out any speculations about progeny expression in the revised version. In the case of *emmpk3*, the RT-qPCR experiments most likely reflect the broad expression of the gene not only in stem cells but also in differentiated cells. The RT-qPCR experiments are now taken up in the M&M section, in the results sections, and as additional Fig. S3.

*4) editorial suggestions*

*L80: the exclusively-> exclusively*

*L263: highest-> the highest*

*L271: 1.462 amino acids -> 1,462 amino acids*

*L437: any of the EmMEKKs -> any of the EmMKKs?*

*L438: with EmMEKK3 -> with EmMKK3?*

*L480: interacted with each other*

*L495-499: 75,9% ->75.9%; 5,0%->5.0% ...*

*Fig. 6B: I suggest using ‘emmpk3-EdU-’ instead of ‘DAPI only’*

All changes have been made according to the reviewers suggestions.

*Reviewer #4: Overall, this study presents novel information on potential signaling pathways in Echinococcus. However, the statement that this study shows that JNK pathways represent novel targets for chemotherapy is not supported by the presented data. I think this statement could be easily removed without impairing the overall impact and importance of this paper.*

As already outlined above, we do not fully agree. Of course, we agree that current lead compounds (such as SP600125) which have been selected among thousands of other compounds to specifically bind to human kinase orthologs are not the drugs of choice against parasites, where they mostly bind with less affinity to the respective kinases. However, this does not mean that it is not possible to find compounds with higher affinity for parasite kinases by rational drug design or maybe even in classical approaches. What it needs is a promising target (important function for the parasite) and structural differences in respective binding pockets between human and parasite orthologs (and it doesn’t even have to be the ATP binding pocket). We think that EmMPK3 fulfils these criteria. At least we showed that a current JNK inhibitor (optimized against human kinases) leads to loss of stem cells in *Echinococcus*, which is of central importance to the biology of this parasite. Furthermore, the fact that there are drugs which bind more efficiently to human kinases than to parasite kinases implies that there are sufficient differences in the binding pockets allowing the identification of binders with higher affinity for parasite kinases. Even if such a compound does not inhibit the parasite kinase more effectively than the human kinase, it could possibly be used for treatment if it just binds less effective to the human kinase. It may be that EmMPK3 is not a first-choice target when compared to other parasite kinases, but this does not disqualify it as a ‘possible’ target. In the revised version of our manuscript, we toned down any statements on the utility of the *Echinococcus* JNK signalling pathway as much as possible (see abstract and introduction), but we nevertheless think that it is legitimate to suggest EmMPK3 as a possible target. We hope the reviewer can accept this.
